# Posterior retroperitoneoscopic adrenalectomy (PRA) in adrenocortical carcinoma (ACC)

**DOI:** 10.1007/s00423-025-03919-x

**Published:** 2025-11-29

**Authors:** Pier Francesco Alesina, Polina Knyazeva, Martin K. Walz

**Affiliations:** 1 Department of Medicine, Universität Witten-Herdecke, Witten, Germany; 2 Department of Surgery and Minimally Invasive Surgery Evang. Kliniken Essen-Mitte, Henricistrasse 92, Essen, 45136 Germany

**Keywords:** Adrenocortical cancer, ACC, Adrenalectomy, Retroperitoneoscopic adrenalectomy, Minimally invasive adrenalectomy, Laparoscopic adrenalectomy

## Abstract

**Introduction:**

Investigating the role of posterior retroperitoneoscopic adrenalectomy (PRA) for the treatment of adrenocortical cancer (ACC).

**Methods:**

Between January 2010 and December 2024, 28 patients (9 men, 19 female) with am mean age of 51.5 ± 19.5 years (range: 1.6–82.3) underwent PRA for primary ACC. Tumor sizes ranged between 3 and 15 cm (mean: 7.3 cm). Hormonal hypersecretion was found in 12 patients. Surgeries were performed in a standardized 3-port technique in prone position. Follow-up (mean: 37.9 months) data could be obtained for 26 patients.

**Results:**

There were 12 right and 16 left adrenalectomies. The mean operating time was 159.2 ± 100.9 min (range: 35–340 min). Seven conversions occurred (25%): five to an open approach and two to a laparoscopic approach. One patient with Cushing‘s syndrome died because of multiple organ failure in the postoperative period (4%). The mean follow-up time was 38.8 ± 35.3 months. Patients with stage I disease demonstrated a 5-year overall survival rate of 100%, whereas patients with stage II and III disease had 3-years survival rates of 64% and 50%, respectively.

**Conclusions:**

The posterior retroperitoneoscopic approach appears feasible in patient with confirmed or suspected ACC and can be proposed in selected cases.

## Introduction

**Fig. 1:**
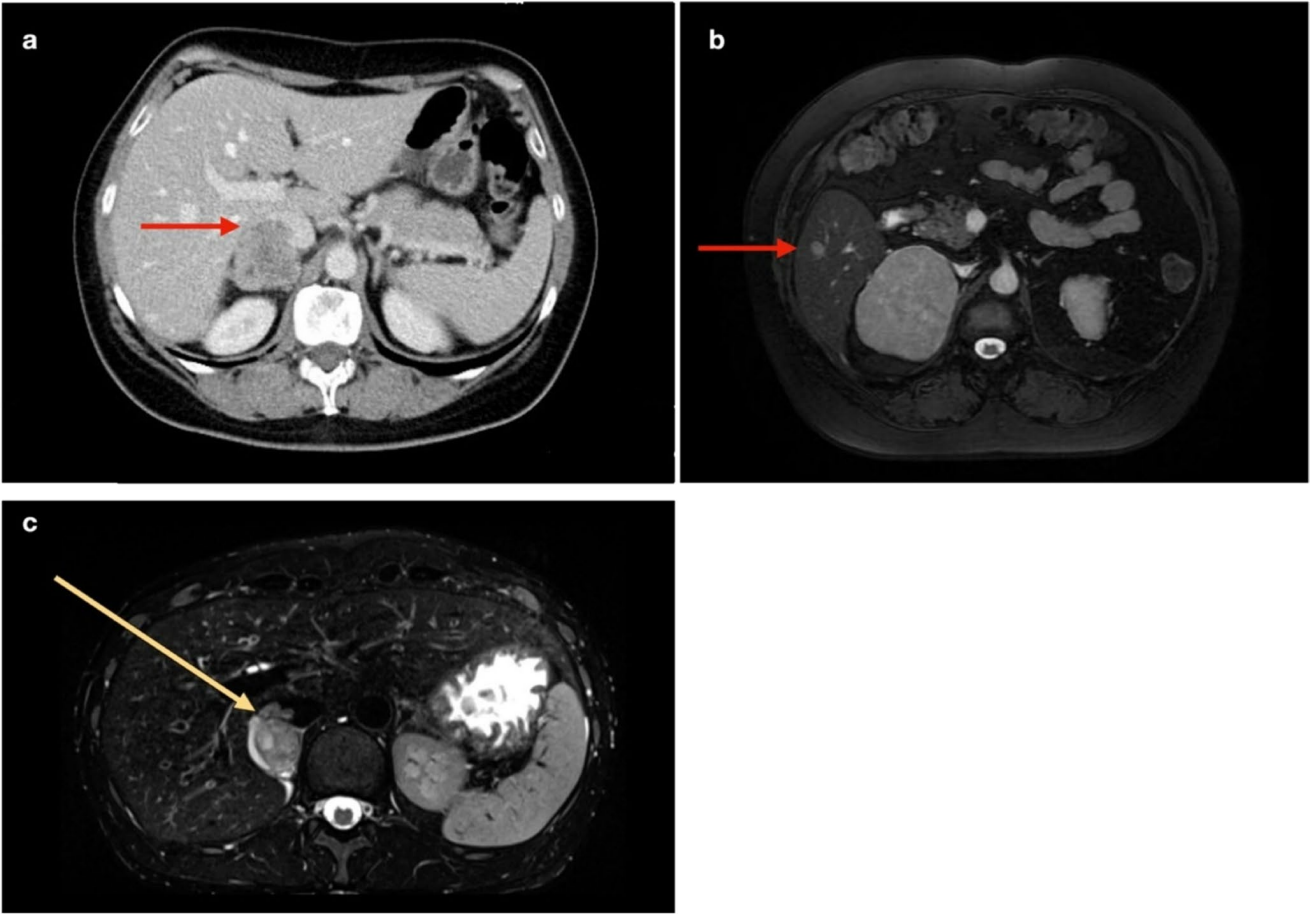
Preoperative imaging of patients with adrenal tumors clearly suspicious for ACC: **a**) computed tomography scan showing the invasion of the inferior vena cava (➔) **b**) magnet resonance imaging with an adrenocortical cancer and a concomitant solitary liver metastasis (➔) **c**) magnet resonance imaging showing ACC with a neoplastic thrombus in the IVC (➔)

Adrenocortical cancer (ACC) is a rare disease. The annual incidence is estimated between 0.5 and 2.0 cases per million persons, with woman more often affected (60%) [1]. The peak of incidence is between 40 and 60 years. To establish the diagnosis of cancer before surgery can be very demanding unless the patient presents with distant metastasis or clear signs of local invasion (Fig. 1). Hormonal hypersecretion, mostly hypercortisolism (either alone or in association with the hypersecretion of other hormones), is diagnosed in approximately 50% of cases. In contrast, generally hormone-inactive tumors are discovered due to symptoms related to mass effect (such as abdominal pain or tumor cachexia) or incidentally in up to 38% of cases [2]. Radical resection is the only curative option and should be considered even in selected patients with distant metastases or recurrent disease since this could yield a survival benefit [3]. The role of minimally invasive surgery for the surgical treatment of adrenocortical cancer is controversial despite there is some evidence that it can achieve equal results to open surgery in term of local control and overall prognosis, at least for patients with European Network for the Study of Adrenal Tumors (ENSAT) stadium I and II disease [4, 5]. Nevertheless, the quality of evidence from these observational studies is very low. A recent metanalysis reviewed 18 studies involving 14,600 patients to compare minimally invasive surgery (MIS) with open adrenalectomy (OA) for adrenocortical carcinoma (ACC). OA generally showed better outcomes in terms of operative time and fewer postoperative complications, while MIS offered shorter hospital stays. Although MIS led to faster recovery, it was associated with higher recurrence rates and earlier recurrences, particularly in stage I–III ACC [6]. The role of the retroperitoneoscopic approach is still uncertain, due to the near absence of outcome data in the literature. For the first time we present a larger series of ACC patients operated by the posterior retroperitoneoscopic adrenalectomy.

## Methods

**Fig. 2:**
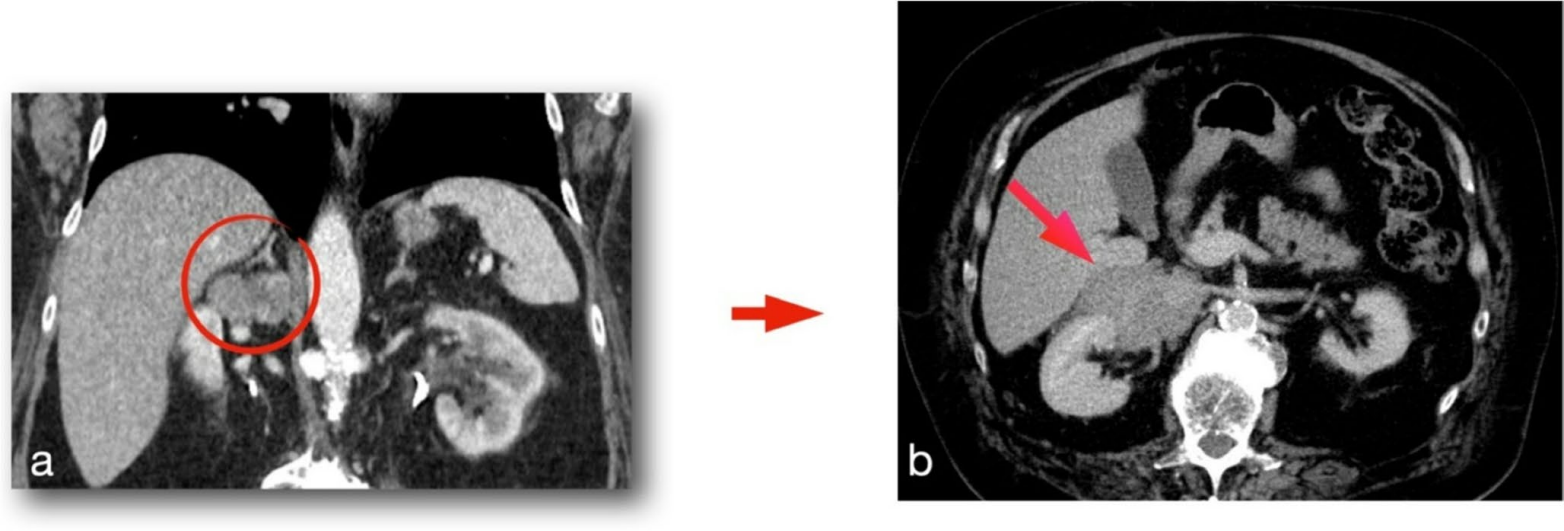
CT scan showing tumor prior to partial adrenalectomy (**a**) for ACC; recurrence developed after 12 months (**b**)

In a period of 15 years (January 2010 - December 2024), 39 patients with primary ACC underwent surgical treatment, two by open approach and 37 by minimally invasive techniques. Of the latter group, 9 patients - operated by transabdominal laparoscopy - were excluded from this study as well as two primary open cases (diameter 15 and 17.5 cm). The core group of this study are 28 patients (9 male, 19 female) who were treated by the posterior retroperitoneoscopic approach on an intention to treat basis. Data were prospectively collected in an institutional database. The mean age of the patients was 51.5 ± 19.5 years (range: 1.6–82.3). Further characteristics are summarized in Table 1. According to the preoperative imaging or intraoperative findings ACC was strongly suspected or diagnosed in 19 cases: 1 case with biopsy (lung) proven Stadium IV ACC, 1 case with liver metastasis, 2 cases with suspected lung metastasis, 6 cases with neoplastic tumor thrombus, 6 cases with suspected lymph node metastasis or tumor growth involving adjacent organs, 2 cases with large tumors and hormonal hypersecretion, but without any other suspicious morphological features on imaging or during surgical exploration (Fig. 2).

**Table 1:** Characteristics of the 28 patients with ACC treated by PRA

Gender	9 M, 19 F
Age (years)	51.5 ± 19.5 (range: 1.6–82.3)
Tumor diameter (cm)	7.3 ± 2.9 (3–15)
Functioning neoplasia Hypercortisolism* *Hyperaldosteronism + hypercortisolism* *Virilizing* Non-funtioning neoplasia	12 8 2 2 16
Preoperative Staging *distant metastasis* *vascular involvement (tumor thrombus*)	1 liver, 3 lung** 6

*one patient with a mild autonomous cortisol secretion (MACS); ** in two patients,* a preoperative CT scan showed a few subcentimetric (< 5 mm) lesions*,* which were confirmed to be metastases (tumor growth) during follow-up*

### Surgical technique

The description of retroperitoneoscopic adrenalectomy, developed by us, has been published in 1995 [7] and the most relevant modifications to the technique later in 2006 [8]. Patients are placed in the prone, half-jackknife position with the lower legs in a 90° angle to the thigh. A rectangular pillow is used between the operating table and the abdomen of the patient to allow the abdominal wall to hang through ventrally; alternatively, a roll may be positioned below the chest and the pelvis. This position creates the optimal working space below the ribs. A 1.5 to 2 cm skin incision is performed at the level of the tip of the 12th rib and the retroperitoneal space is opened by blunt and sharp dissection with scissors. A finger is inserted into the retroperitoneum to widen the space and palpate the 11th rib from inside. A 5 mm port is than inserted just below the tip of the 11th rib under digital control. A blunt trocar with an inflatable balloon and an adjustable sleeve (Blunt Tip^®^, Medtronic, Minneapolis, USA) is introduced into the initial incision site and blocked. The CO2 insufflation is started with a pressure of 20 mmHg and can be increased up to 30 mmHg according to the characteristics of the patient (degree of obesity and amount of retroperitoneal fatty tissue) and to the dimension of the tumor. The working space is created by opening Gerota’s facia and pushing the retroperitoneal fatty tissue ventrally and caudally. By this manoeuvre the kidney and the adrenal gland are visualized. A third trocar (5–10 mm in diameter) is inserted medially below the 12th rib under visual control paying attention to avoid lesions the subcostal nerve running parallel to the rib. The dissection starts on the lateral aspect of the upper pole of the kidney and aims to mobilize the kidney and free it from the adhesions to the retroperitoneal fatty tissue. The kidney is retracted caudally and medially to expose the lower pole of the adrenal gland; according to the different anatomical position of the left and right adrenal gland the mobilisation of the kidney should be more extensively performed on the left side. The dissection is then continued medially. On the right side the vena cava (IVC) is visualized and the retrocaval dissection is performed until the adrenal vein is reached; here small arteries running horizontal behind the IVC are generally encountered and divided. On the left side the adrenal vein is isolated medially by completing the dissection of the lower pole of the adrenal. When performing adrenalectomy by suspicious of ACC a modification of the technique can be considered to extent the resection to the regional lymph nodes with the aim of improving local control and increasing the likelihood of achieving an R0 resection. On the right side the renal artery and vein are identified, and the dissection line follows the renal vessels until reaching the inter-aortocaval region to dissect the fatty tissue including the paraadrenal and inter-aortocaval lymph nodes. The posterior wall of the vena cava is completely exposed, and the lymphadenectomy can be continued until reaching the right crux. At the level of the upper third of the adrenal gland the short adrenal vein is than isolated and divided. On the left side the renal artery is identified and dissected free until reaching its origin at the aorta. The left paraaortal fatty tissue including the lymph nodes is removed and the aorta is visualized till the level of the left crux. The left adrenal vein running into the renal vein is automatically identified as well as the inferior diaphragmatic vein. The division of the adrenal vein is performed by energy device (Ligasure^®^, Medtronic, Minneapolis, USA). The cranial dissection represents the next step of the procedure and is followed by the dissection of the anterior Gerota layer on the right side and the lamina between the pancreas and the adrenal on the left side. For radicality reasons these layers can be removed “en-bloc” with the tumor exposing the right posterior segments of the liver on the right side and the pancreas and splenic vessels on the left side. Opening of the peritoneal layer at this step of the procedure does not reduce significantly the operating space allowing a safe completion of the procedure. For the same reason, the renal capsule, particularly at the upper pole, can be removed along with the tumor, exposing the renal parenchyma and avoiding nephrectomy in most cases, as direct infiltration of the renal parenchyma is rare. The specimen is placed in a retrieval bag and pulled through the middle incision; in bag fragmentation is performed, if required, to facilitate the extraction. Suction drains are generally not used.

### Follow-up

To analyze the oncological outcome we used the European Network for the Study of Adrenal Tumors (ENSAT) staging system: Stage I ACC ≤5 cm in size and confined to the adrenal gland, without disease in nearby lymph nodes or distant sites (N0 and M0); Stage II ACC was defined as an N0M0 tumor > 5 cm confined to the adrenal gland; Stage III ACC was defined as a tumor with the disease in nearby nodes (N1), infiltration of surrounding tissue, or vascular extension without evidence of distant metastasis; Stage IV ACC was defined as a metastatic tumor (M1). Follow-up data were obtained by in-hospital oncological database or contacting the general physician and/or the patients.

### Statistical analysis

Data are expressed as mean ± standard deviation. Collected data were processed (Prism 10, GraphPad^®^). Disease-free and overall survivals were estimated from Kaplan–Meier methods.

## Results

There were 13 right and 15 left adrenalectomies. Retroperitoneoscopic adrenalectomy was mainly performed according to the standard 3-port-technique. In two patients the adrenal tumor was removed in single access technique (SARA) [9]: one was a 49-year-old female with an incidentaloma on the right side and an ipsilateral nephroptosis. The non-functioning 5 cm ACC was not diagnosed or suspected prior surgery. The other patient (2-year-old boy) suffered from a virilizing left-sided ACC (diameter 5 cm). The pediatric operation was modified with reduced CO2 insufflation pressure (16 mmHg). All patients underwent total adrenalectomy with one exception, a patient with a 5 cm eccentric right adrenal incidentaloma which was removed by partial adrenalectomy (because of in-bag fragmentation the R-status could not be evaluated at final histology).

Mean operating time including conversions was 159.2 ± 100.9 min (range: 35–340 min) and 123.9 ± 85.8 min (range: 30 − 3 00 min) without conversions. Mean operating time did not differ between right side (135.8 ± 94.8 min) and left side (160.6 ± 97.7 min) [*p* = 0.5]. Estimated median intraoperative blood loss was neglectable (< 10 ml; range: 0–2000 ml). Nevertheless, two patients required blood transfusions due to significant intraoperative bleeding. No intraoperative deaths occurred. The conversion rate was 25%, including five cases converted to laparotomy (18%) and two to transabdominal laparoscopy (7%). Notably, five out of the seven conversions were on the left side. In three cases, the reason for conversion was related to the difficulty of dissection and tumor size (> 10 cm). In one case, an initial conversion to laparoscopy was followed by open surgery due to suspected liver infiltration. In the remaining three cases, conversion was required due to the presence of a tumor thrombus—two involving the inferior vena cava (IVC) and one involving the left renal vein.

In the first patient, a right-sided tumor measuring 13 cm was found to have severe adhesions to the IVC and a neoplastic thrombus, requiring tangential resection of the vein and concomitant nephrectomy. In the second patient, the neoplastic thrombus extended from the left renal vein up to the IVC; this patient also had concomitant liver metastasis confined to the right hepatic lobe. The procedure began with retroperitoneoscopic mobilization of the adrenal gland, including dissection of the renal hilum. Intraoperative ultrasound revealed a neoplastic thrombus reaching the confluence of the left renal vein into the IVC. Thrombectomy of the IVC and en bloc resection of the adrenal tumor with left nephrectomy were completed via open surgery. The right-sided liver resection, initially planned as a two-stage procedure, was then performed in the same session. In the third case, which involved a left-sided renal vein thrombus, emergency conversion was necessary due to incomplete vascular control and intraoperative bleeding. The reason for conversion from the retroperitoneoscopic to the laparoscopic approach was the difficult dissection associated with elevated level of intraoperative pCO2. Thrombectomy was performed by the retroperitoneoscopic approach in three cases (two on the right side and one on the left side) after obtaining full vascular control of the IVC and the left renal vein, respectively. A lymphadenectomy was performed in 8 cases, including two cases of conversion. The number of removed lymph nodes ranged from 1 to 18. Five patients (18%) were admitted to the intensive care unit, postoperatively. Postoperative complications included: (1) one superficial surgical site infection of the median laparotomy; (2) one bleeding on the 5th day after surgery requiring retroperitoneoscopic reoperation; (3) one death on the 2nd postoperative day due to multiple organ failure in a patient with severe Cushing’s syndrome. The results are summarized in Table 2.

**Table 2:** Perioperative results of PRA for 28 patients with primary ACC

Operating time (minutes)	159.2 ± 100.9 (range: 35–340)
Extended resection *liver resection* *nephrectomy* *renal capsula resection* *thrombectomy* *lymphadenectomy* *muscle resection (psoas)*	2* 2* 3 6** 8 1
Median blood loss (ml)	5 ml (range: 0–2000)
Conversions *from PRA to open* *from PRA to laparoscopy*	7 5 2
Histopathological data *R0/Rx* *N+*	4/24 4/8
Tumor stage (ENSAT) *Stage I* *Stage II* *Stage III* *Stage IV*	3 14 6 5
Postoperative complications *wound infection* *bleeding* *death (multiple organ failure)*	1 1 1

*after conversion; **three after conversion (in one case the neoplastic thrombus was diagnosed intraoperatively)

There was no case of macroscopical evidence of incomplete resection, intraoperatively. Due to the routine praxis of tumor fragmentation a fully evaluation of the microscopical resection margin status was not possible in most of the cases; an R0 resection was histologically confirmed in four cases after conversion to open surgery. The Weiss score was not routinely assessed for histological examination. Ki67 immunohistochemical analysis was available in 21 cases (mean value 32.7 ± 17; range: 10–80%). Seven patients with overt Cushing’s syndrome received postoperative oral cortisone therapy; intraoperative and/or intravenous cortisone substitution was not used. The mean postoperative hospital stay was 3.5 ± 2.3 days (range: 2–14 days). Excluding conversions, mean postoperative hospital for PRA was 3.4 ± 2.5 days (range: 2–11 days).

According to the ENSAT tumor classification there were 3 (10.6%) patients with a stage I disease, 14 (50%) with a stage II, 6 (21.4%) with a stage III, and 5 (18%) with a stage IV. Information about the postoperative oncological treatment was available for all but 3 patients (2 in stage II and 1 in stage III disease). Patients at stage I received no further treatment after surgery. Mitotane was the standard treatment for patients at stage II; in one case a concomitant local radiation therapy was performed; the patient after partial adrenalectomy refused adjuvant therapy. Patients at stage III and IV received mitotane; a combination of mitotane and platinum-based chemotherapy was administered in 6 cases. Following thrombectomy of the vena cava and an uneventful postoperative recovery, mitotane treatment was discontinued in one patient a few months after surgery due to adverse effects. One patient at stage IV died three months after surgery due to cardiac insufficiency and pneumonia, with progressive lung metastases, after being deemed unfit for adjuvant treatment.

The mean follow-up time was 38.8 ± 35.3 months. Of the 28 patients, 13 (46.5%) are alive. One patient was lost to follow-up. Two patients died 16 and 74 months after surgery, although in both cases it remains unclear whether the deaths were related to ACC. Ten patients are disease-free, including both patients after single-port surgery. One patient with a 7 cm right-sided tumor associated to hyperpcortisolism and hyperaldosteronism developed multiple distant metastasis; she is still alive 82 months after surgery. The second patient underwent right-sided adrenalectomy with thrombectomy of the vena cava. She subsequently developed retroperitoneal lymph node metastases and bilateral pulmonary metastases, all of which were successfully resected. Recently, immunotherapy was initiated due to recurrence in the lower abdomen. The third patient developed lung metastasis twelve months after adrenalectomy. During the follow-up, four patients underwent reoperations (14%). In one case a systematic retroperitoneoscopic paraaortic lymphadenectomy was performed two months after the primary surgery. Metastases were found in 3 of 17 lymph nodes. This patient died 6 years later. A second patient, who developed a port-site metastasis, required reoperation 10 months after adrenalectomy. She died 48 months after adrenalectomy due to distant metastases. In the third patient with locoregional recurrence after partial adrenalectomy and Cushing’s syndrome a two-step retroperitoneal debulking was performed to improve the hormonal hypersecretion. This patient received afterwards best supportive palliative therapy and died 15 months after the first operation. The latter patient underwent adrenalectomy and thrombectomy of the vena cava. She developed metastasis in the inter-aorto-caval lymph nodes 15 months after the primary surgery and underwent a systematic retroperitoneoscopic lymphadenectomy; five metastases were found upon final histology. Three months later, a PET-CT scan revealed a paravertebral loco-regional recurrence, which was again resected using the retroperitoneoscopic approach.

**Fig. 3:**
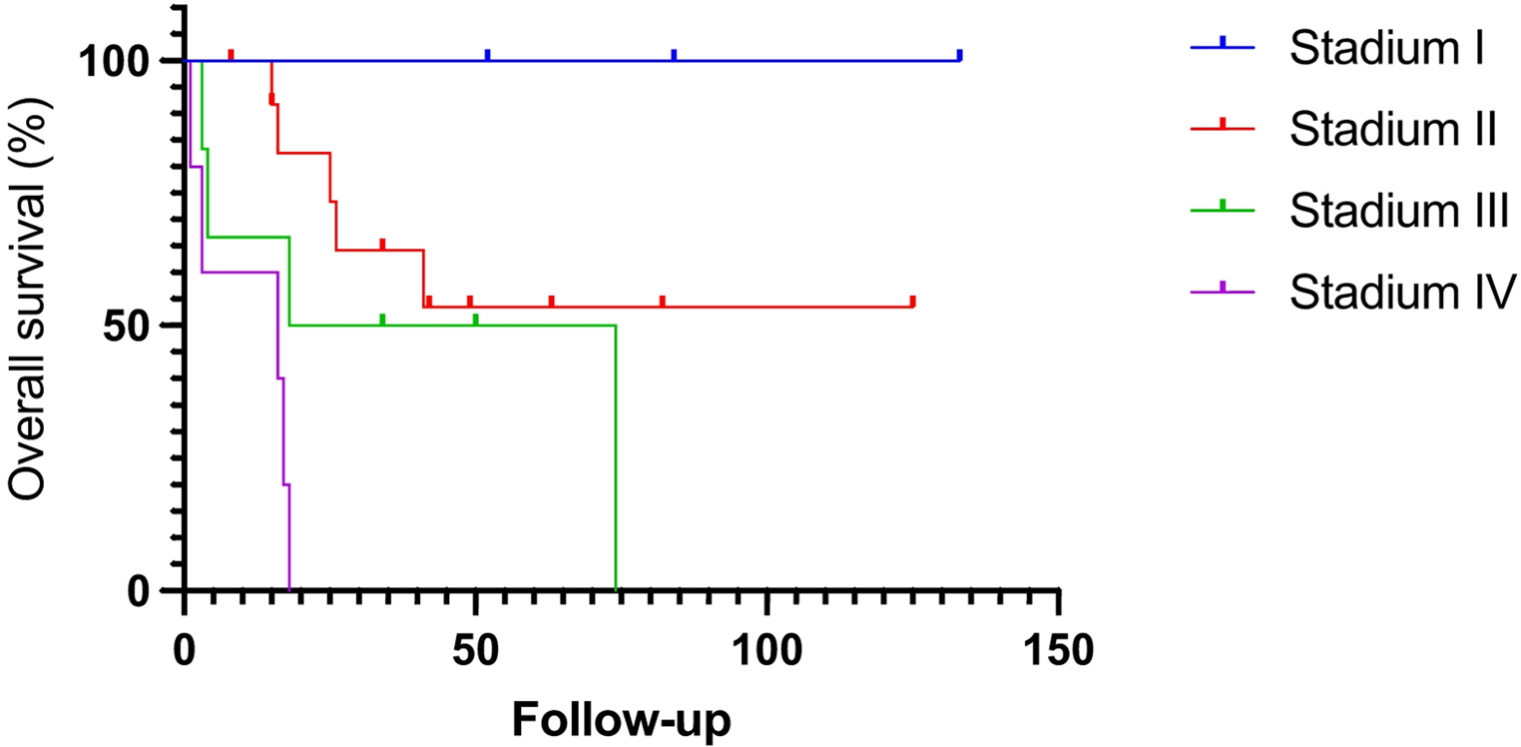
Overall survival of 27 patients with ACC stage I-IV removed by PRA *(Kaplan-Meier)*

**Fig. 4:**
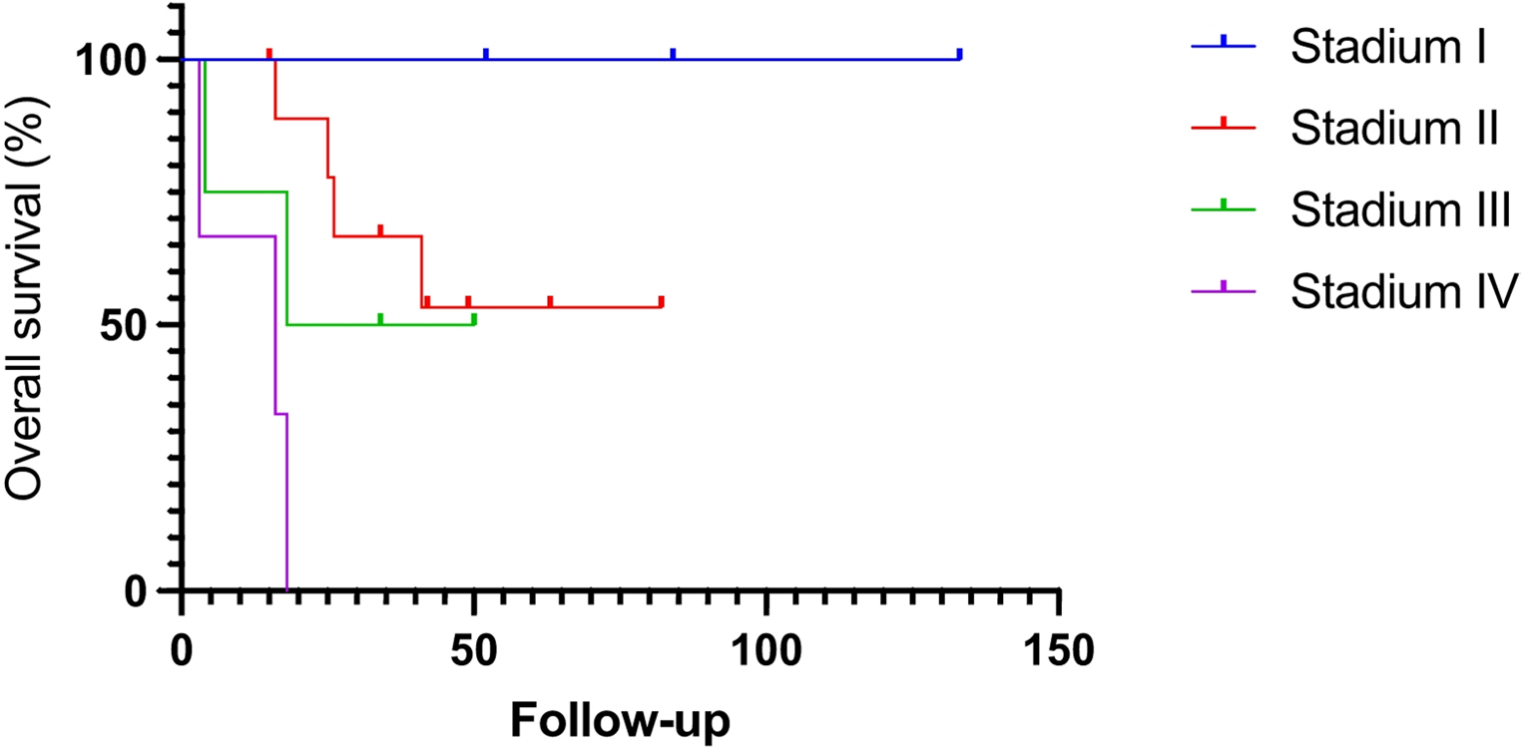
Overall survival of 20 patients with ACC stage I-IV removed by PRA, excluding conversions. *(Kaplan-Meier)*

Overall survival according to the intention-to-treat analysis, including all patients regardless of conversion, is shown in Fig. 3. Subsequently, survival data excluding converted cases are presented to highlight outcomes in patients who underwent a completed minimally invasive procedure (Fig. 4). All patients with ENSAT stage I disease are alive, with follow-up periods ranging from 52 to 133 months. In patients with stage II and III disease, the 3-year overall survival rates are 64% and 50%, respectively. The estimated 5-year survival rates are 53% for stage II and 50% for stage III. Notably, one patient with stage II disease remains alive at the last follow-up (82 months after surgery) despite the presence of multiple metastases. Two patients died 16 and 74 months after surgery, although in both cases it remains unclear whether the deaths were related to ACC. All five stage IV patients died within two years after adrenalectomy.

## Discussion

The use of minimally invasive techniques for the surgical treatment of adrenocortical cancer is still a matter of debate. The European Society of Clinical Endocrinology (ECE) in collaboration with the ENSAT recommend open surgery as the standard surgical approach for confirmed or highly suspected ACCs; however, for tumors < 6 cm without any evidence of local invasion, minimally invasive adrenalectomy can be considered [3]. Though lack of clear evidence, experts believe that six adrenalectomies per year is sufficient in benign adrenal diseases, but >20 annual cases are desirable for surgery in ACCs [3]. The paper presents for the first time the results of PRA for ACC. In the light of the complexity of the disease with large dimension of the tumors and potential presence of vascular and adjacent organ involvement, the operation can be safely performed with a conversion rate of about 20%. Latter does not differ from transperitoneal laparoscopic adrenalectomy. Delozier et al. described a conversion rate to open surgery of 19.4% in 196 patients [10]. Risk factors for conversions were larger tumors (9 cm) and the right-side cases. On multivariable Cox regression analysis, tumor size >6 cm and right-sided location were confirmed as independent predictors of conversion during attempted minimally invasive adrenalectomy. Conversion significantly reduced overall survival at two years and was associated with a higher risk of R1 resection. Additionally, R1 resection rates were higher in cases requiring conversion compared to planned open adrenalectomies. These results suggest that maintaining a low threshold for conversion to open surgery before risking tumor capsule damage may be advisable [10]. In our series, seven procedures were converted to either open (*n* = 5) or laparoscopic (*n* = 2) surgery due to dissection difficulties related to tumor size, suspected liver infiltration (which was not confirmed by final histology), or the presence of tumor thrombus in the inferior vena cava (IVC) or left renal vein. In four patients an R0 resection without capsule infraction was confirmed by histology; in one case with neoplastic thrombus a margin-free resection could not be confirmed at final pathology (Rx). Four out of five patients who were converted to open surgery died during the follow-up period. In two cases, conversion to laparoscopy was performed. One patient is alive at the last follow-up, 125 months after surgery; the second developed lung metastasis 12 months after adrenalectomy.

In principle, minimally invasive adrenalectomy has demonstrated superiority to open surgery in terms of blood loss, recovery time, and postoperative pain [11]. These items are secondary in cancer patients as cure is the main aim. Recent retrospective studies show equal survival in laparoscopic and open surgery based on patients’ selection and extended experience, at least for patients with localized (stage I–II) ACC [4, 5, 12, 13]. Others reported an improved local control [14] or less positive tumor margins [15] in open surgery without demonstrating improved survival. In the present series, the three-year survival rate is 100% for patients with stage I disease, whereas it is 64% and 50% for those with stage II and III disease, respectively. This finding differs from data reported in the literature, where higher survival rates for stage II disease have been observed. Several factors may explain this discrepancy. First, our study reports overall survival, not disease-specific survival. For instance, one patient with stage II disease died 16 months after surgery, but it remains unclear whether the cause of death was tumor-related. Second, both a patient who developed port-site metastasis and another who underwent partial adrenalectomy were classified as stage II and died of tumor-related causes 15 and 48 months after primary surgery, respectively.

**Fig. 5:**
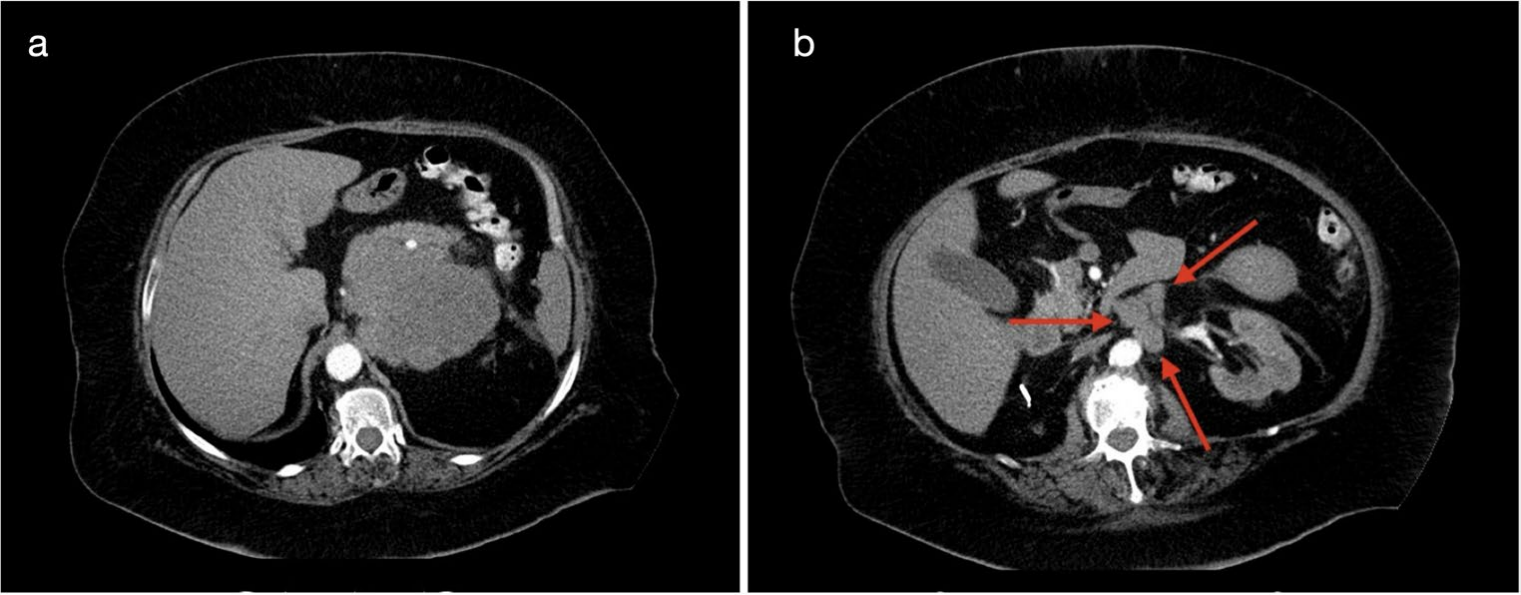
Computed tomography of a female patient with left-sided ACC **a**) primary tumor (9 cm), **b**) paraaortic lymph nodes metastases (arrows)

ACC surgery requires expertise in both adrenal and oncological surgery as well as tremendous skills and advanced technology in minimally invasive surgery. Based on the experience of our first PRA case in ACC with early locoregional recurrence due to unrecognized tumor capsula rapture, we stopped minimally invasive surgery in this disease. A restart was mainly possible by the groundbreaking improvements of imaging (2006 HD, 2016 4 K) allowing higher levels of precise dissection. Without doubt, proper patient’s selection is another key to success. Patients with localized tumors (stage I and II) seem to be best candidates for minimally invasive surgery. In our approach, oncologic safety remains the primary goal, and the decision to proceed with minimally invasive adrenalectomy is never based on cosmetic outcomes, but rather on the conviction that, in selected cases, it can offer the best balance between radical resection and patient benefit. Importantly, macroscopic complete resection (R0) continues to serve as the most critical indicator of surgical success, particularly given the possible limitations of pathological margin assessment in large adrenal tumors. Furthermore, a minimally invasive approach may be used for initial exploration, and conversion to open surgery should not be viewed as a failure, but rather as a strategic decision to ensure oncologic completeness. The choice of surgical approach is individualized, based on tumor characteristics, preoperative imaging, and the surgeon’s expertise. In our experience, patients with localized disease (stage I and II) are the most suitable candidates for minimally invasive adrenalectomy, provided that surgical radicality can be assured. For laparoscopic adrenalectomy a limit of 6–10 cm has been proposed [12]. Tumors between 8 and 10 cm appear to be borderline for the posterior approach due to limited working space. Based on our nearly thirty years of experience, tumor size, patient dimensions, and the amount of retroperitoneal fatty tissue surrounding the neoplasm must be considered for safe surgery. Locoregional lymph node metastases are not a contraindication for PRA, as retroperitoneal lymphadenectomy can be performed alongside resection of the primary tumor. The diagnostic and therapeutic role of routine lymphadenectomy (LND) is not completely clarified as it is used in a wide range of 10–30% of cases [16–18]. In a recent meta-analysis LND improves survival in stage I-III but not in stage IV [19]. The number of resected lymph nodes may be important as more than five reduce the risks of local recurrence and disease-related death [19]. From our point of view, left paraaortal and right interaortocaval and paracaval lymphadenectomy extended to the hilum of the kidney and to the infrarenal region is feasible by the retroperitoneoscopic approach. Indeed, this technique offers a direct access to the retroperitoneum avoiding the mobilization of the intraabdominal organs and their possible injury. In cases with extended pathological (especially contralateral interaortocaval) lymph nodes minimally invasive techniques should be abandoned (Fig. 5).

The need for vascular control is not per se a limit of the retroperitoneoscopic adrenalectomy. In our series, 6 patients with intravenous tumor thrombus are included. In two right-sided cases the thrombus involved the inferior vena cava (IVC). In four left-sided tumors, the thrombus reached the renal vein (*n* = 3), in one case the IVC. Conversion to open surgery was required in three cases. A fully endoscopic exposure of the IVC and renal vessels was possible in the remaining three allowing minimally invasive thrombectomy and vessels reconstruction by the posterior approach as already published in our case report in 2021 [20]. For this purpose, laparoscopic reusable clips (Aesculap^®^ Bulldog Clips, Braun, Germany) are necessary to occlude the venous segment between the renal veins and the hepatic veins. This is certainly a demanding procedure that requires specialized skills and should be reserved for expert surgeons. In this subgroup, one patient demonstrated a disease-free postoperative course with no recurrence after 68 months of follow-up. A second patient developed locoregional lymph node and later lung metastasis requiring reoperation. All patients received adjuvant mitotane therapy, with one also receiving additional conventional chemotherapy. These data correspond to the survey conducted by the European Society of Endocrine Surgeons, which includes cases operated on via open surgery [21].

The need for multivisceral resection prompted conversion in four of our patients. In two cases, the presence of tumor thrombus required concomitant nephrectomy, and one patient with liver metastasis also underwent a right hepatectomy. Conversion to an anterior open approach facilitated safe vascular control and complete resection. However, preoperative assessment of local infiltration remains challenging. The primary site of infiltration is Gerota’s fascia rather than the pancreas, liver, or kidney parenchyma itself. Notably, the kidney is generally not extensively infiltrated, even when the tumor capsule is in contact with the renal surface, causing slight displacement of the organ. A broad decapsulation of the upper renal pole can be technically advantageous, allowing for safer manipulation around the lower portion of the tumor. Minor bleeding from the renal parenchyma can be effectively managed with diathermy. On the right superior region, the peritoneum behind the liver may serve in the same way. Furthermore, it has been shown, even if on a small number of patients, that en-bloc resection of stage II-ACCs and kidney does not improve survival [22]. In rare cases, if required, it is technically possible to perform a nephrectomy by the retroperitoneoscopic approach and on the right side wedge resection of the liver [23].

The study has certain limitation due to its retrospective design and the small number of patients. Our pathological reports do not mention scores or completeness of surgery as almost all tumors are fragmented in the retrieval bags. Scores have been replaced by a simplified method to distinguish between benign and malignant adrenocortical neoplasia, according to the presence of tumor necrosis [24].

In conclusion, the posterior retroperitoneoscopic approach is feasible and safe for selected patients with ACC and limited disease and can be proposed as an adequate alternative to open or laparoscopic adrenalectomy tumors ≤ 8–10 cm. Lymphadenectomy is possible in more advanced tumors in highly selected patients. Avoiding the opening of the peritoneal cavity can potentially eliminate the risk of peritoneal seeding (none in our series) after surgery. To confirm the results of this study in the setting of a prospective randomized trial seems unrealistic considering the rarity of the disease. Nevertheless, collecting more data about minimally invasive treatment of ACC will improve our knowledge and hopefully improve the prognosis of the disease whose treatment should be always managed by a multidisciplinary team including surgeons, endocrinologist, and oncologist.

## Data Availability

The datasets generated and/or analysed during the current study are available from the corresponding author on reasonable request.

## References

[1] Kerkhofs TM, Verhoeven RH, Van der Zwan JM, Dieleman J, Kerstens MN, Links TP, Van de Poll-Franse LV, Haak HR (2013) Adrenocortical carcinoma: a population-based study on incidence and survival in the Netherlands since 1993. Eur J Cancer (Oxford England: 1990) 49(11):2579–2586. 10.1016/j.ejca.2013.02.034 10.1016/j.ejca.2013.02.034 23561851

[2] Puglisi S, Calabrese A, Ferraù F, Violi MA, Laganà M, Grisanti S, Ceccato F, Scaroni C, Dalmazi GD, Stigliano A, Altieri B, Canu L, Loli P, Pivonello R, Arvat E, Morelli V, Perotti P, Basile V, Berchialla P, Urru S, Terzolo M (2023) New findings on presentation and outcome of patients with adrenocortical cancer: results from a national cohort study. J Clin Endocrinol Metab. 10.1210/clinem/dgad199 10.1210/clinem/dgad199 37022947

[3] Fassnacht M, Dekkers OM, Else T, Baudin E, Berruti A, de Krijger R, Haak HR, Mihai R, Assie G, Terzolo M (2018) European society of endocrinology clinical practice guidelines on the management of adrenocortical carcinoma in adults, in collaboration with the European network for the study of adrenal tumors. Eur J Endocrinol 179(4):G1–G46. 10.1530/EJE-18-0608 30299884 10.1530/EJE-18-0608

[4] Brix D, Allolio B, Fenske W, Agha A, Dralle H, Jurowich C, Langer P, Mussack T, Nies C, Riedmiller H, Spahn M, Weismann D, Hahner S, Fassnacht M, German Adrenocortical Carcinoma Registry Group (2010) Laparoscopic versus open adrenalectomy for adrenocortical carcinoma: surgical and oncologic outcome in 152 patients. Eur Urol 58(4):609–615. 10.1016/j.eururo.2010.06.024 20580485 10.1016/j.eururo.2010.06.024

[5] Porpiglia F, Fiori C, Daffara F, Zaggia B, Bollito E, Volante M, Berruti A, Terzolo M (2010) Retrospective evaluation of the outcome of open versus laparoscopic adrenalectomy for stage I and II adrenocortical cancer. Eur Urol 57(5):873–878. 10.1016/j.eururo.2010.01.036 20137850 10.1016/j.eururo.2010.01.036

[6] Giordano A, Feroci F, Podda M, Botteri E, Ortenzi M, Montori G, Guerrieri M, Vettoretto N, Agresta F, Bergamini C (2023) Minimally invasive versus open adrenalectomy for adrenocortical carcinoma: the keys surgical factors influencing the outcomes-a collective overview. Langenbecks Arch Surg 408(1):256. 10.1007/s00423-023-02997-z 37386332 10.1007/s00423-023-02997-z

[7] Walz MK, Peitgen K, Krause U, Eigler FW (1995) Die dorsale Retroperitoneoskopische Adrenalektomie–eine neue operative technik [Dorsal retroperitoneoscopic adrenalectomy–a new surgical technique]. Zentralbl Chir 120(1):53–58 7887040

[8] Walz MK, Alesina PF, Wenger FA, Deligiannis A, Szuczik E, Petersenn S, Ommer A, Groeben H, Peitgen K, Janssen OE, Philipp T, Neumann HP, Schmid KW, Mann K (2006) Posterior retroperitoneoscopic adrenalectomy–results of 560 procedures in 520 patients. Surgery 140(6):943–950. 10.1016/j.surg.2006.07.039 17188142 10.1016/j.surg.2006.07.039

[9] Walz MK, Alesina PF (2009) Single access retroperitoneoscopic adrenalectomy (SARA) - one step beyond in endocrine surgery. Langenbecks Arch Surg 394(3):447–450. 10.1007/s00423-008-0418-z 18784938 10.1007/s00423-008-0418-z

[10] Delozier OM, Stiles ZE, Deschner BW, Drake JA, Deneve JL, Glazer ES, Tsao MW, Yakoub D, Dickson PV (2021) Implications of conversion during attempted minimally invasive adrenalectomy for adrenocortical carcinoma. Ann Surg Oncol 28(1):492–501. 10.1245/s10434-020-08824-9 32656720 10.1245/s10434-020-08824-9

[11] Brunt LM, Doherty GM, Norton JA, Soper NJ, Quasebarth MA, Moley JF (1996) Laparoscopic adrenalectomy compared to open adrenalectomy for benign adrenal neoplasms. J Am Coll Surg 183(1):1–10 8673301

[12] Donatini G, Caiazzo R, Do Cao C, Aubert S, Zerrweck C, El-Kathib Z, Gauthier T, Leteurtre E, Wemeau JL, Vantyghem MC, Carnaille B, Pattou F (2014) Long-term survival after adrenalectomy for stage I/II adrenocortical carcinoma (ACC): a retrospective comparative cohort study of laparoscopic versus open approach. Ann Surg Oncol 21(1):284–291. 10.1245/s10434-013-3164-6 24046101 10.1245/s10434-013-3164-6

[13] Gaillard M, Razafinimanana M, Challine A, Araujo RLC, Libé R, Sibony M, Barat M, Bertherat J, Dousset B, Fuks D, Gaujoux S (2023) Laparoscopic or open adrenalectomy for stage I-II adrenocortical carcinoma: a retrospective study. J Clin Med 12(11):3698. 10.3390/jcm12113698 37297891 10.3390/jcm12113698 PMC10253560

[14] Wu K, Liu Z, Liang J, Tang Y, Zou Z, Zhou C, Zhang F, Lu Y (2018) Laparoscopic versus open adrenalectomy for localized (stage 1/2) adrenocortical carcinoma: experience at a single, high-volumecenter. Surgery 164(6):1325–1329. 10.1016/j.surg.2018.07.026 30266443 10.1016/j.surg.2018.07.026

[15] Miller BS, Ammori JB, Gauger PG, Broome JT, Hammer GD, Doherty GM (2010) Laparoscopic resection is inappropriate in patients with known or suspected adrenocortical carcinoma. World J Surg 34(6):1380–1385. 10.1007/s00268-010-0532-2 20372905 10.1007/s00268-010-0532-2

[16] Icard P, Goudet P, Charpenay C, Andreassian B, Carnaille B, Chapuis Y, Cougard P, Henry JF, Proye C (2001) Adrenocortical carcinomas: surgical trends and results of a 253-patient series from the French association of endocrine surgeons study group. World J Surg 25(7):891–897. 10.1007/s00268-001-0047-y 11572030 10.1007/s00268-001-0047-y

[17] Nilubol N, Patel D, Kebebew E (2016) Does lymphadenectomy improve survival in patients with adrenocortical carcinoma? A population-based study. World J Surg 40(3):697–705. 10.1007/s00268-015-3283-2 26510563 10.1007/s00268-015-3283-2

[18] Reibetanz J, Jurowich C, Erdogan I, Nies C, Rayes N, Dralle H, Behrend M, Allolio B, Fassnacht M, German, ACC study group (2012) Impact of lymphadenectomy on the oncologic outcome of patients with adrenocortical carcinoma. Ann Surg 255(2):363–369. 10.1097/SLA.0b013e3182367ac3 22143204 10.1097/SLA.0b013e3182367ac3

[19] Hendricks A, Müller S, Fassnacht M, Germer CT, Wiegering VA, Wiegering A, Reibetanz J (2022) Impact of lymphadenectomy on the oncologic outcome of patients with adrenocortical carcinoma-a systematic review and meta-analysis. Cancers (Basel) 14(2):291. 10.3390/cancers14020291 35053453 10.3390/cancers14020291 PMC8774191

[20] Walz MK, Jongekkasit I, Alesina PF (2021) Retroperitoneoscopic adrenalectomy in adrenocortical carcinoma with tumor thrombus in inferior vena cava: one step further in minimally invasive endocrine surgery. VideoEndocrinology. 10.1089/ve.2020.0202

[21] Mihai R, Iacobone M, Makay O, Moreno P, Frilling A, Kraimps JL, Soriano A, Villar del Moral J, Barczynski M, Durán MC, Sadler GP, Niederle B, Dralle H, Harrison B, Carnaille B (2012) Outcome of operation in patients with adrenocortical cancer invading the inferior Vena cava–a European society of endocrine surgeons (ESES) survey. Langenbeck’s Archives Surg 397(2):225–231. 10.1007/s00423-011-0876-6 10.1007/s00423-011-0876-6 22134748

[22] Porpiglia F, Fiori C, Daffara FC, Zaggia B, Ardito A, Scarpa RM, Papotti M, Berruti A, Scagliotti GV, Terzolo M (2016) Does nephrectomy during radical adrenalectomy for stage II adrenocortical cancer affect patient outcome? J Endocrinol Invest 39(4):465–471. 10.1007/s40618-015-0422-4 26694705 10.1007/s40618-015-0422-4

[23] Alesina PF, Walz MK (2019) A new minimally invasive approach to the posterior right segments of the liver: report of the first two cases. J Laparoendosc Adv Surg Tech A 29(7):943–948. 10.1089/lap.2018.0809 30912692 10.1089/lap.2018.0809

[24] Walz MK, Metz KA, Theurer S, Myland C, Alesina PF, Schmid KW (2020) Differentiating benign from malignant adrenocortical tumors by a single morphological parameter-a clinicopathological study on 837 adrenocortical neoplasias. Indian J Surg Oncol 11(4):705–710. 10.1007/s13193-020-01205-4 33281410 10.1007/s13193-020-01205-4 PMC7714795

